# Formulation of a fish feed for goldfish with natural astaxanthin extracted from shrimp waste

**DOI:** 10.1186/s13065-016-0190-z

**Published:** 2016-07-19

**Authors:** W. K. O. V. Weeratunge, B. G. K. Perera

**Affiliations:** Department of Chemistry, Faculty of Science, University of Colombo, Colombo 3, Sri Lanka

**Keywords:** Astaxanthin, Antioxidant capacity, Antibacterial activity, Shrimp waste

## Abstract

**Background:**

Astaxanthin is a xanthophyll carotenoid, which exhibits many important biological activities including a high degree of antioxidant capacity (AOC) and antibacterial activity, hence has a significant applicability in food, pharmaceutical and cosmetic industries. An attempt was made towards optimization of astaxanthin extraction conditions using three different extraction conditions and a solvent series, from uncooked, cooked and acid-treated shrimp waste, which is a readily available and cheap source of the pigment. The astaxanthin extracts were analyzed by comparing their UV–visible absorbance spectra and thin layer chromatograms with a standard astaxanthin sample. The percentage of astaxanthin in each crude sample was determined using the Beer–Lambert law. The Folin–Ciocalteu assay and the disk diffusion assay were used to investigate the antioxidant capacities and antibacterial activities of extracted astaxanthin samples respectively. The extracted astaxanthin was incorporated into fish feeds to test its ability to enhance the skin color of goldfish.

**Results:**

The best astaxanthin percentage of 68 % was observed with the acetone:ethyl acetate (1:1) solvent system facilitated by maceration of cooked and acid treated shrimp, whereas the best crude yield of 33 % was found to be in the acetone extract of the acid-treated shrimp sample. The highest AOC of 65 µg pyrogallol equivalents/mg was observed for the EtOAc extract obtained by maceration of acid-treated shrimp waste. The highest AOC by sonication and soxhlet extraction methods were also obtained with the EtOAc solvent. The extracts exhibited antibacterial activity against four selected bacterial strains. The newly formulated astaxanthin enriched fish feed was economical and indicated a significant improvement of the skin color and healthiness of goldfish compared to the control feeds.

**Conclusion:**

Biologically active astaxanthin can be successfully extracted from shrimp waste in higher percentages. The extraction technique and the solvent used to extract astaxanthin from shrimp waste should be decided depending on the desired outcome and application of astaxanthin. Moreover, the novel astaxanthin enriched fish feed formulated during this study was found to effectively enhance the skin color of goldfish within 10 days, a much shorter feeding period compared to previously reported feeding periods in similar studies.

**Graphical abstract:**
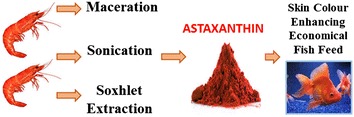
Formulation of a skin color enhancing fish feed for ornamental fish using crude astaxanthin extracted from shrimp waste

**Electronic supplementary material:**

The online version of this article (doi:10.1186/s13065-016-0190-z) contains supplementary material, which is available to authorized users.

## Background

**Fig. 1:**
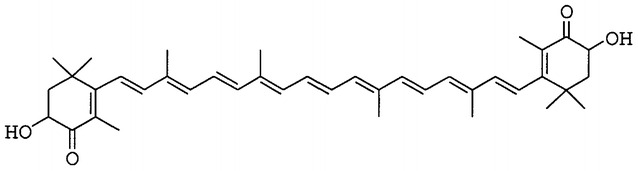
Structure of astaxanthin

Astaxanthin (Fig. 1) is a tetraterpene carotenoid pigment, mainly obtained from seafood and algae [1]. It imparts bright red colour to crustacean exoskeleton [2], to salmon flesh [3] and to the feathers of flamingos [4]. The presence of hydroxyl groups at the terminal rings of astaxanthin categorizes it under xanthophylls, and results in a slight polarity despite of the long conjugated hydrocarbon chain.

The high antioxidant capacity [5] and the antimicrobial properties [6] of the pigment make it applicable in many of the industrial fields, such as cosmetic, pharmaceutical and food industry [7]. Astaxanthin possesses a significantly high free radical scavenging capacity, compared to other carotenoids and vitamin E, thereby has the ability to prevent severe health problems including inflammation, age related macular degeneration, cancers and atherosclerosis [7, 8].

Astaxanthin can be either extracted from natural sources such as red algae, *Haematococcus pluvialis* [9] and crustacean exoskeletons or they can be chemically synthesized using the Wittig reaction between a dialdehyde and a phosphonium salt, dienolether condensation or isomerization of lutein to zeaxanthin followed by its oxidation to a ketone [10]. In spite of natural astaxanthin consisting of (3S, 3′S) as the only isomer, synthetic astaxanthin results in a mixture of isomers in a ratio of (3S, 3′S):(3R, 3′S):(3R, 3′R) 1:2:1 [11]. Furthermore, during chemical synthesis, the production of *cis* isomers demotivate the use of chemically synthesized astaxanthin. [11] Therefore, extraction of natural astaxanthin from red algae, crustaceans and red yeast, *Phaffia rhodozyma* is preferred in the astaxanthin industry [12]. However, yeasts produce only the (3R, 3′R) isomer [10], which is found to be the isomer with the least antioxidant capacity (AOC) [11]. Furthermore, maintenance of algal cultures to get astaxanthin requires careful control of the lighting and temperature and demands for much time, specialized skills, specific equipment and special care to avoid pathogenic diseases [13]. Considering all these factors, shrimp waste seems to be an easy passage to extract astaxanthin in an economically beneficial manner.

Shrimp processing waste is the largest industrial fish waste in many of the countries all over the world, thus giving rise to several environmental problems [5]. They can act as a preferable substrate for the growth of pathogens as well [14]. This study reports the results from an investigation of the optimum astaxanthin extraction conditions from differently processed shrimp waste using three different extraction techniques and a series of solvents. This research could possibly be a solution for the fore-stated environmental problem as well. During this study, efforts were made to study the bioactivities of crude astaxanthin extracts as that may help to bypass an additional processing step that could be beneficial in an industrial level to reduce cost and time.

Astaxanthin pigment is found in the integument of the ornamental fish [15]. Carotenoids cannot be synthesized de novo by the fish and therefore, need to be obtained via food. It has also been reported that dietary lipids could improve the skin colour of fish [16]. This study reveals the ability of crude astaxanthin extracts which contain natural lipid substances extracted along with the pigment to be incorporated into fish feeds towards enhancing the skin color of goldfish (*Carassius auratus*). Furthermore, effect of incorporation of dietary coconut oil and soya oil in these astaxanthin enriched fish feed are also reported.

## Experimental

### Reagents, chemicals, instruments and organisms

Acetone, EtOAc, hexane and methanol were general purpose reagents. Analytical grade silica gel 70–230 mesh (Sigma Aldrich, India) was obtained from Department of Chemistry, University of Colombo, Sri Lanka. Mueller–Hinton Agar was purchased from Royal Surgicals, Colombo, Sri Lanka and Gentamycin was purchased from Union Chemists, Colombo, Sri Lanka. Coconut oil and soya oil were purchased from a supermarket, Colombo, Sri Lanka. The standard astaxanthin algal sample was purchased from a United State supplier through e-bay (Source—*Haematococcus pluvialis*, purity—about 85 %).

UV–visible spectrophotometers—Jasco v 560 and Genesys 10S, analytical balance—Ohrus PA313, visible range spectrophotometer—spectrum Shanghai 721E, oven—Memmert Beschickung 100–800, Autoclave machine—ALP Co. Ltd KT 30SD, Shaker—Taitec BR-40L, Sonicator bath—Bandelin Sonorex Super RK 1028 CH, Laminar flow—BIOBASE.

Bacterial cultures were obtained from Department of Chemistry, University of Colombo and the goldfish were purchased from Oasis Aquarium, Kiribathgoda, Sri Lanka.

Differently processed shrimp waste was used to extract astaxanthin using three different extraction techniques; maceration, sonication and Soxhlet extraction and five different solvents; acetone, ethyl acetate (EtOAc), methanol, hexane and acetone:EtOAc (1:1) mixture.

### Preparation of shrimp waste for extraction

Raw shrimp waste was washed with water and sundried. Then it was powdered by grinding. Heat treated shrimp waste was prepared by heating the sundried shrimp waste to around 80 °C in a pan and grinding into a powder. Acid treated shrimp waste was prepared by dipping the sundried and powdered raw shrimp waste in 4 M HCl. (10 mL of HCl for 1 g of shrimp). Then the sample was immersed in a 70 °C water bath for 2 min [7] The acid treated shrimp waste was collected by filtration. All the samples were refrigerated until further use.

### Extraction and characterization of astaxanthin

#### Maceration

Weights of 2 g of heat treated shrimp shell waste were dipped in 30 mL of acetone, EtOAc (EtOAc), acetone:EtOAc (1:1) mixture, methanol and hexane separately in amber colour bottles. These bottles were shaken overnight using an electrical shaker. After 20 h of maceration, the samples were taken out of the shaker, filtered and the solvents were evaporated. The same extraction procedure was carried out with 0.7 g of acid treated shrimp waste or 0.6 g of raw shrimp shell waste using 15 mL of acetone, EtOAc and acetone:EtOAc (1:1) mixture as solvents.

#### Sonication

Masses of 0.5 g of heat treated shrimp waste were subjected to sonication for 4 h with 15 mL of acetone, EtOAc, acetone:EtOAc (1:1) mixture, methanol and hexane as solvents. Then the samples were filtered, concentrated.

#### Soxhlet extraction

A portion of 1 g of heat treated shrimp waste was placed in the thimble of a Soxhlet apparatus. A volume of 100 mL of hexane, EtOAc or methanol were added into its round bottom flask and astaxanthin was extracted into the solvent by refluxing for 3 h. Finally, the extract was filtered, concentrated.

All the samples were covered with aluminium foil and stored until further use. The crude astaxanthin yields were calculated for all the extracts. UV—visible spectra of the crude astaxanthin samples were compared with a standard astaxanthin spectrum. The percentage of astaxanthin in each extract was calculated using Beer–Lambert law and the absorbance at 470 nm (ɛ as 206 L/g/cm) [17].

Thin layer chromatograms of crude astaxanthin samples displaying the highest crude percentage yield, highest astaxanthin percentage and the highest antioxidant capacity were developed using acetone:hexane (3:7) [18] mixture as the mobile phase. The resultant band patterns were compared with a standard astaxanthin sample originated from *Haematococcus pluvialis.*

### Antioxidant capacity by Folin–Ciocalteu assay

A volume of 0.100 mL of each astaxanthin sample, dissolved in methanol was mixed with 2 mL of 2 % (w/v) sodium bicarbonate and was incubated at room temperature for 2 min [19]. Then, 0.100 mL of the prepared Folin–Ciocalteu reagent was added into each sample. The samples were incubated for 30 min under dark conditions [19]. The absorbance of each extract was measured at 750 nm using a spectrophotometer. The AOC of each astaxanthin extract was determined using a standard curve of pyrogallol. [AOC is given in pyrogallol equivalents (PGE)]. The assays were carried out in triplicate.

### Antibacterial activity by disk diffusion assay

During the antibacterial studies, the astaxanthin extracts, which showed the highest antioxidant capacity, highest crude yield and the highest astaxanthin percentage were dissolved in acetone and were used in the disk diffusion assay. Sterilized 6 mm filter paper disks were dipped in acetone—diluted astaxanthin extracts with known concentrations (50, 100, 150, and 200 mg/mL). The disks were dried and were placed on the spread plates prepared with *Staphylococcus aureus* (ATCC 25923), *Salmonella typhimurium* (ATCC 14028), *Bacillus cereus* (ATCC 11778) and *Escherichia coli* (ATCC 35218). A 25 μL/mL gentamycin solution and acetone were used as the positive and negative controls respectively. The plates containing the disks were incubated overnight at 37 °C. Then the average diameter of the inhibition zones were measured and recorded. The assays were carried out in triplicate. Finally, the lowest concentration of the astaxanthin extracts that can inhibit bacterial growth was recorded.

### Effectiveness of astaxanthin incorporated fish feeds towards enhancing skin colour and healthiness of goldfish

Three sets of goldfish (*Carassius auratus*) were fed for 20 days with three separate fish feeds prepared as indicated in Table 1. The healthiness of fish groups were monitored in terms of the mortality percentages during the course of feeding. At the end of the feeding period, a visual sensory evaluation was carried out using a group of 26 individuals in the age group of 20–60 years.

**Table 1:** Compositions of the fish feeds

Feed	Astaxanthin (%)	Coconut oil (%)	Shrimp waste (%)	Gelatin (%)	Bread crumbs (%)
A1	1	1	–	1	97
S1	–	1	99	–	–
C1	–	–	–	–	100

### Determination of a suitable dietary oil to be incorporated into the fish feed

Similarly, four sets of fish were fed with the fish feeds indicated in Table 2. The fish were fed for 10 days and a visual sensory evaluation was carried out with 35 evaluators. Healthiness of fish was also monitored during this period of study. The healthiness of fish groups were measured in terms of mortality percentages during the course of study.

**Table 2:** Compositions of different fish feeds used to investigate the impact of coconut oil

Feed	Astaxanthin (%)	Coconut oil (%)	Gelatin (%)	Bread crumbs (%)
A1	1	1	1	97
A2	1	–	1	98
C2	–	1	1	98
C3	–	–	1	99

A similar experiment was carried out using soya oil instead of coconut oil. Three sets of fish were fed for 10 days with the feeds indicated in the Table 3 below. A visual sensory evaluation was done at the end of the 10 day feeding period with 35 evaluators.

**Table 3:** Compositions of different fish feeds used to investigate the impact of soya oil

Feed	Astaxanthin (%)	Soya oil (%)	Gelatin (%)	Bread crumbs (%)
A3	1	1	1	97
A2	1	–	1	98
C4	–	1	1	98

The necessary ethical clearance was obtained from the relevant authorities for the use of ornamental fish for this study (Registration No: ERC IOBSL 126 05 15).

## Results and discussion

### Characterization of astaxanthin extracts

According to the results indicated in Table 4, acetone and methanol resulted the highest crude yields for astaxanthin extracts independent of the extraction technique. The highest crude yield of 33 % was observed for the acid treated shrimp waste sample macerated using acetone. Heat treated shrimp macerated in methanol has also provided with a reasonable crude percentage yield of 32 %. The highest percentage of astaxanthin (68 % of crude weight) was obtained with both acid treated and heat treated shrimp waste, when extracted with Acetone:EtOAc (1:1) mixture by maceration. This observation could be attributed to the degradation of the carotenoprotein complexes upon heat or acid treatment, releasing the tightly bound astaxanthin pigments out. Thus, heat or acid treatment could be carried out to obtain extracts with greater astaxanthin percentages. The highest AOC of 65 μg PGE/mg was recorded for the acid treated shrimp waste macerated using EtOAc, followed by an AOC of 56 μg PGE/mg for the raw shrimp macerated in EtOAc. EtOAc extracts had the higher AOC regardless of the extraction technique. However, maceration was selected to be the best extraction method to obtain astaxanthin extracts with much greater AOCs.

**Table 4:** Summary of variation of selected properties of selected extracts with extraction technique

Solvent	Percentage crude yield (%)					Percentage astaxanthin yield (%)					AOC (µg PGE/mg)				
	M			Sn	Sx	M			Sn	Sx	M			Sn	Sx
	R	H	A	H	H	R	H	A	H	H	R	H	A	H	H
Acetone	26	5	33	9	–	7	62	62	22	–	4	14	2	14	–
Acetone: EtOAc (1:1)	8	3	11	8	–	4	68	68	62	–	5	14	10	12	–
EtOAc	1	4	1	6	3	39	12	29	56	24	56	4	65	20	28
Hexane	–	3	–	4	6	–	44	–	8	11	–	9	–	12	10
Methanol	–	32	–	17	6	–	28	–	41	28	–	2	–	10	10

*M* maceration, *Sn* sonication, *Sx* soxhlet extraction, *R* raw shrimp waste, *H* heat treated shrimp waste, *A* acid treated shrimp waste

During the antibacterial studies carried out with the astaxanthin extracts with highest crude yield, astaxanthin yield and AOC capacity, it was observed that there was no direct correlation between the antibacterial activity and the astaxanthin percentage of the shrimp extracts (see Additional file 1: Table S1).

### Effectiveness of the fish feeds prepared with extracted astaxanthin

Feeding the fish with required nutrients plays a crucial role in enhancing the skin colour and healthiness [20] of the fish. Carotenoids such as astaxanthin are often included in fish feed in various forms to obtain attractive body colours and other benefits [15]. Other than incorporation of synthetic or purified astaxanthin into fish feeds, powdered shrimp waste is more commonly used as the astaxanthin source in fish feeds [21, 22]. Use of crude pigment extracts instead of purified or synthetic astaxanthin could be of great benefit, due to the presence of additional proteins, carbohydrates and fatty acids in the crude extracts, thereby fulfilling the nutritional requirements of the fish being fed.

According to the visual sensory evaluations (Table 5) carried out to explore the effectiveness of the form of astaxanthin added to the fish feeds towards enhancing the skin color of goldfish, it was found out that fish feed prepared with extracted astaxanthin (A1) could significantly improve the skin colour of goldfish within 20 days of feeding compared to the fish fed with raw shrimp waste powder (S1) as the astaxanthin source or the fish group fed with the feed lacking astaxanthin (C1).

**Table 5:** Investigation of the effectiveness of the form of astaxanthin incorporated into fish feed

Statement about the intensity of skin colour^a^	Percentage of evaluators in agreement (%)
Fish fed with A1 (with extracted crude astaxanthin) were the darkest in skin colour	42
Fish fed with S1 (with shrimp waste) were the darkest in skin colour intensity	27
Both sets of fish fed with A1 and S1 were the darkest and had similar skin colour intensity	12
Fish fed with the C1 (no astaxanthin) were the darkest in skin colour	8
Any other answer	11

^a^Darker skin colour = high intensity of red colour/brighter red colour

The important role of astaxanthin towards improving the skin colour of goldfish was supported by 81 % of the evaluators who have agreed that incorporating any form of astaxanthin could enhance the red colour of goldfish skin. Among these evaluators, 42 % clearly agreed that incorporation of crude astaxanthin extract obtained from shrimp waste was much more efficient towards fish skin colour enhancement compared to the direct use of shrimp waste as the astaxanthin source (27 %), whereas 12 % of these evaluators mentioned that extracted astaxanthin and shrimp waste itself are of equal capacity towards improving the skin color of goldfish.

Previously published research has indicated the ability of coconut oil to improve the absorption of carotenoids in Mongolian gerbil fish [16]. Olive oil and fish oil have also been shown as potential candidates for the dietary lipid source. However, the use of these oils in a fish feed is not economically beneficial [23]. Olive oil and fish oil are mainly composed of long chain polyunsaturated fatty acids and omega three fatty acids therefore, are not much beneficial in the aspect of nutrition [24]. There is a current tendency of people to use soya oil all over the world, because of its significance in bearing omega 6 fatty acids which are claimed to be healthier [25]. During this study, the suitability of soya oil to serve as a dietary lipid source was explored with comparison to coconut oil. Similar to previous observations, 89–90 % of the evaluators confirmed that astaxanthin can improve the skin colour of goldfish (Table 6).

**Table 6:** Evaluation of the effectiveness of dietary oil in fish feed

Statement about the intensity of skin colour^a^	Percentage of evaluators in agreement (%)	
	Coconut oil	Soya oil
Fish fed with feeds containing astaxanthin with oil (A1 or A3) were the darkest in colour	33	83
Fish fed with feeds containing astaxanthin without oil (A2) were the darkest in colour	57	6
Both fish groups fed with astaxanthin containing feeds (A1, A2 and A3) were darker and similar in colour intensity	0	0
Fish fed with control feeds with oil but no astaxanthin (C2 and C4) were the darkest in colour	8	11
Fish fed with controls without both astaxanthin and oil (C3) were the darkest in colour	2	–
Any other answer	0	0

^a^Darker skin colour = high intensity of red colour/brighter red colour

**Fig. 2:**
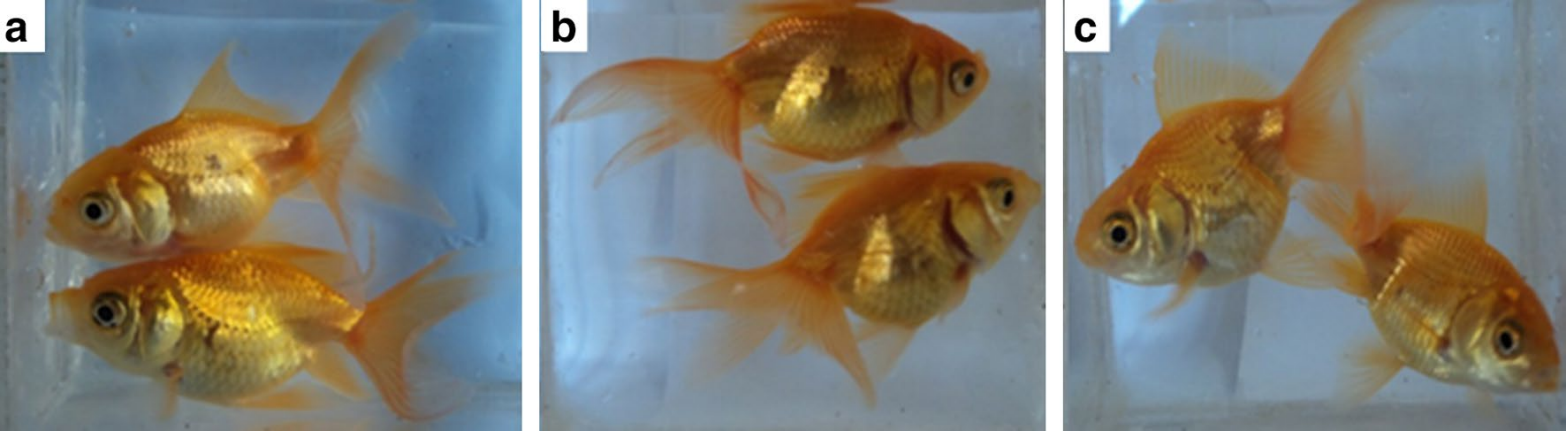
Goldfish samples used for the visual sensory evaluation **a** Fish fed with A2, **b** Fish fed with A3, **c** Fish fed with C4

The colour enhancement due to astaxanthin was greatly facilitated by the addition of soya oil to the fish feed and this was indicated with a majority of evaluators (83 %) clearly agreeing that the fish feed A3 containing soya oil resulted in darker coloured fish compared to the fish fed with feed A2 lacking soya oil (6 %) (Fig. 2). However, the contribution from coconut oil was not as significant as from soya oil. Only 33 % agreed that fish fed with feed A1 containing coconut oil resulted in darker fish colour compared to the 57 % of evaluators who agreed otherwise.

Coconut oil is composed of medium chain length saturated fatty acids that improve the solubility of carotenoids inside the body of the fish [16]. In addition to improving the solubility, soya oil consisting of unsaturated long chain fatty acids facilitates the micellarization and chylomicron packaging of carotenoids as well [16]. These additional benefits of soya oil might be contributing to the improved effectiveness of astaxanthin enriched fish feed towards colour enhancement.

In addition to the skin colour enhancement, improvement of the healthiness of goldfish was also monitored during their feeding period with astaxanthin enriched feed (Table 7).

**Table 7:** Mortality percentages of fish groups during feeding periods

Feed	Mortality percentage
A1	0
S1	33
C1	50

Fish fed with astaxanthin containing feeds A1 and S1 indicated low mortality percentages compared to the fish group fed with C1, the feed lacking astaxanthin. This indicates the impact of astaxanthin towards improving the healthiness of fish, possibly due to its capacity as a powerful antioxidant agent or antibacterial agent. The high survival rate of the fish fed with A1 compared to the group fed with S1 helps to conclude that the incorporation of extracted astaxanthin into fish feed is a better option than the direct usage of shrimp waste as the astaxanthin source.

## Conclusions

Maceration of either acid or heat treated shrimp waste in acetone:EtOAc (1:1) solvent mixture resulted the crude extract with the highest astaxanthin percentage of 68 %. The highest AOC was obtained for the astaxanthin extract obtained by maceration of acid treated shrimp in EtOAc. The crude astaxanthin extracts also displayed varying degrees of antibacterial activity against four selected bacterial strains independent of the extraction conditions used. Incorporation of 1 % of the crude astaxanthin extract into a newly formulated fish feed along with 1 % of soya oil significantly improved the skin colour and healthiness of goldfish within 10 days of feeding. The novel fish feed formulated using soya oil during this study indicated higher efficiency towards enhancing the skin pigmentation of goldfish compared to fish feed prepared using coconut oil as indicated in previous studies. Incorporation of crude astaxanthin extracts into the fish feed also contribute towards cost reduction compared to the usage of totally purified or synthetic astaxanthin.

## Additional file

10.1186/s13065-016-0190-z Minimum active astaxanthin concentrations against selected species for selected crude extracts.

## Abbreviations

AOC: antioxidant capacity
A1: fish feed composed of crude astaxanthin and coconut oil in trial 1 and 2
S1: fish feed composed of shrimp waste and coconut oil in trial 1
C1: non-astaxanthin feed without coconut oil in trial 1
A2: fish feed with crude astaxanthin and without a dietary oil in trial 2 and 3
C2: non-astaxanthin feed with coconut oil in trial 2
C3: non-astaxanthin feed without coconut oil in trail 2
A3: fish feed with astaxanthin and soya oil in trial 3
C4: non-astaxanthin feed with soya oil in trial 3
R: raw shrimp waste
H: heat treated shrimp waste
A: acid treated shrimp waste
Sn: sonication
Sx: soxhlet extraction
M: Maceration
