# Supplementary material for: Formulation of a fish feed for goldfish with natural astaxanthin extracted from shrimp waste
Source: Chem Cent J. 2016 Jul 19;10:44. doi: 10.1186/s13065-016-0190-z (PMC4952235; doi:10.1186/s13065-016-0190-z)
Supplement: Supplementary file 1 — 10.1186/s13065-016-0190-z Minimum active astaxanthin concentrations against selected species for selected crude extracts. [file 13065_2016_190_MOESM1_ESM.docx]

Additional File 1 Minimum active astaxanthin concentrations against selected species for selected crude extracts

| Bacterial strain | Minimum active astaxanthin concentration against the species (mg/mL) | | |
| --- | --- | --- | --- |
|  | Acid treated Macerated Acetone extract (Highest crude yield) | Heat treated Macerated Acetone: EtOAc extract (Highest astaxanthin yield) | Acid treated Macerated EtOAc extract (Highest AOC) |
| *Staphylococcus aureus* | 100 | 200 | 100 |
| *Salmonella typhimurium* | 100 | 150 | 100 |
| *Bacillus cereus* | 150 | 200 | 150 |
| *Escherichia coli* | 200 | 200 | 200 |
